# How Did CNBSS Influence Guidelines for So Long and What Can That Teach Us?

**DOI:** 10.3390/curroncol29060313

**Published:** 2022-05-30

**Authors:** Shushiela Appavoo

**Affiliations:** Department of Radiology and Diagnostic Imaging, University of Alberta, 2A2.41 WMC 8440-112 Street, Edmonton, Alberta, AB T6G 2B7, Canada; sappavoo@ualberta.ca

**Keywords:** mammography, mammographic screening, randomization, expertise, guidelines, evidenc, epistemic trespassing, evidence-based medicine

## Abstract

The biased randomization and other quality concerns about the Canadian National Breast Screening Studies (CNBSS) were documented and criticized for decades, even by several individuals very close to the research. CNBSS were the outlier studies among several RCTs of the era and yet were given equal weighting and occasionally higher importance than the remainder of the canon of mammography RCTs. These studies have had an ongoing influence on subsequent evidence review, guideline formation, and, ultimately, patient access to screening. This article explores possible reasons for the ongoing inclusion of CNBSS in the body of mammography screening evidence, discusses the lack of expertise in critical healthcare guideline processes, and, ultimately, suggests several actions and reforms.

## 1. Introduction

*People talk about evidence as if it could really be weighed in scales by a blind Justice. No man can judge what is good evidence on any particular subject, unless he knows that subject well.*

George Eliot (Mary Ann Evans), Middlemarch

Recent eyewitness accounts [1,2,3] of the Canadian National Breast Screening Studies (CNBSS) have finally confirmed what was long suspected about the biased allocation of symptomatic women in the screening arm of the trials. Clinical breast examination was performed before allocation at 14 out of 15 study sites, and witnesses confirm that in at least some of those sites, symptomatic women were preferentially placed in the mammography arm of the study. Additionally, symptomatic patients were recruited for mammographic assessment within the screening arm of the studies. This skewed the data, resulting in more late-stage cancers and deaths for women undergoing mammography than for women allocated to the non-mammography arm.

The results of CNBSS have created ongoing doubt about the benefit of screening mammography, particularly in the 40–49 age group, where there was little other research at the time. CNBSS have been used in the formulation of guidelines worldwide for decades, including the Canadian Task Force on Preventive Health Care (CTFPHC) [4], the US Preventive Services Task Force (USPSTF) [5], European Commission [6], World Health Organization (WHO) [7], and more. Yet, early on, CNBSS received extensive criticism about many aspects of implementation.

The volunteer-based recruitment for CNBSS was fundamentally different from the remainder of the mammography randomized controlled trials (RCTs), which were population-based. As a result of the volunteer recruitment, there were high levels of contamination in CNBSS. Women allocated to the control arm of the trial, but who had volunteered because they were motivated to screen, were more likely to seek mammography outside the trial [8,9]. Difficulties in recruitment were even acknowledged by one of the studies’ authors [10], lending plausibility to the eyewitness accounts of CNBSS accepting referrals of symptomatic patients. 

The study data also pointed to non-random allocation of women between the mammography and usual care arms. In CNBSS1 [11], equal numbers of women were randomized to either mammography or usual care. Twenty-four late-stage cancers were noted in total. Of these, 19 were allocated to mammography, and 5 were allocated to usual care, a 380% difference. As an expected consequence of this overwhelming imbalance, the 7-year follow-up study demonstrated that 38 women had died in the mammography arm, and 28 women had died in the usual care arm. A study of enrollees at the Winnipeg study site demonstrated that eight out of nine enrolled women, who had prior billing records for breast cancer (an exclusion criterion), were allocated to the mammography arm of the trial, further suggesting non-random allocation [12].

Several articles were published criticizing the allocation and skewed statistics, including a calculation that the imbalance of late-stage cancers between the mammography and non-mammography arms could have occurred randomly only 3.3 times out of 1000 [13,14,15]. The eyewitness accounts of flawed randomization confirm that which has been evident in the data since early in the studies.

Unfortunately, very few RCTs specifically addressed the 40–49 age group, and, therefore, CNBSS1 has had a large influence on breast screening recommendations for women in this age range. The statistical problems are obvious, so why was this study not excluded by the statistics and epidemiology experts writing guidelines? Several factors may be at play and point to a larger problem with the practical application of evidence-based medicine.

## 2. The Flaws in CNBSS Ignored

CNBSS were criticized long before the results were published. The problematic implementation was questioned by external reviewers [16] and the studies’ own physicists [17]. There were even attempts to explain away the implausible and unprecedented early finding of excess deaths in the screening arm of the trial [18]. No other study among the eight mammography RCTs ever demonstrated this finding. This lack of reproducibility, alone, should have resulted in skepticism about the results.

Early criticism of CNBSS was so widespread that a forensic assessment was published in 1997. This review was limited. Only 3 of 15 sites were assessed, and, importantly, the study staff was not interviewed at that time, despite this step being mandated in the study design [19]. In fact, the authors of this assessment suggested a confirmation bias in their own article, stating that, “We believe that there would be two advantages to publishing the 7-year follow-up data … First, this criticism of the study would end…”. Unfortunately, the quality of the forensic assessment was not questioned, and this study appeased those who would use CNBSS for future guidelines [20,21].

Interestingly, a recent modelling study used only CNBSS as the source material, choosing to focus on the outlier study and ignoring the remaining body of RCTs that converged on a significant benefit to screening [22]. The 2016 USPSTF guideline article went so far as to state, “[Malmo Mammographic Screening Trial I and the Canadian National Breast Screening Study 1 and 2] provided the least-biased estimates” [5].

Despite problematic recruitment and glaring statistical imbalances, recognized decades ago, CNBSS continue to influence research, guidelines, and worldwide guideline-based policy around breast screening. In Canada, CTFPHC guidelines strongly influence many provincial Clinical Practice Guidelines, which may, in turn, define patient access to screening through physician referral practices, programmatic screening structure, and billing restrictions.

How does a study that has been plagued by extensive international criticism over its design and skewed data manage to continue influencing recommendations for decades?

## 3. Evidence-Based Medicine, Evidence Review, and Guidelines Methodology

As a result of the evidence-based medicine movement, modern guidelines hinge on evidence review. This is performed by specialized bodies that conduct systematic searches for literature, decide which evidence is appropriate to include in the review, and then synthesize the data, often building upon older evidence reviews of the same topic. While this appears to be an ideal and objective way to expertly handle large amounts of research and perform the complicated statistical and epidemiological calculations involved, evidence review has some limitations.

Content experts have little to no substantial influence on evidence review. For example, no radiologist is included on the list of contributors for the 2018 CTFPHC breast screening evidence review [23].

Many members and frequently the chairs of evidence review and guideline bodies are non-physicians, and, thus, clinical experience and context are minimized. The continued inclusion of CNBSS in guideline evidence reviews is a stark example of the peril of minimizing content expert input. Had content experts been allowed appropriate input into the guideline processes, the well-documented imbalance in late-stage cancers and other significant problems with implementation could have been made clear to the reviewers.

Evidence review is expensive, and evidence reviews are built upon older reviews to save time and money. Once an error has been made, however, it may be perpetuated by copying that error into future versions of the review. This is what is known in radiology as “alliterative error”, which is the tendency to perpetuate prior errors, particularly when the previous report has been viewed before assessment of the images—or evidence—one has been tasked with assessing [24].

In addition to the evidence review process, guideline methodology and guideline oversight are problematic. While the evidence review tool, GRADE [25], recommends including observational data, the evidence review team and guideline bodies may choose to ignore this, as seen in the 2018 CTFPHC breast screening recommendations [4]. In this guideline, the evidence review included only randomized controlled trials, largely performed between the 1960s and the 1980s, for the calculation of benefits. Decades of more recent screening program data were ignored. The largest observational study of screening program data in the world is known as the Pan Canadian Study, published in 2014 [26]. This demonstrates an overall mortality benefit of 40% for women attending the screening. In the 40–49 age group, this mortality benefit is even higher at 44%. This study is missing from the 2018 CTFPHC breast screening guideline references, and it is even absent from the list of excluded evidence [27]. It is difficult to explain the fact that landmark Canadian evidence is missing from a Canadian evidence review, but the near-complete absence of content experts from the evidence review process may contribute to this oversight.

The AGREEII [28] guideline development and appraisal instrument recommends the inclusion of content experts and patients as advisors on guideline panels, as do many other guideline methodology recommendations [29,30]. Again, however, oversight into the actual guideline process is lacking, and the systematic exclusion of content experts and patients from panels such as CTFPHC’s has largely gone unnoticed.

## 4. Epistemic Trespassing

When is an expert not an expert? Perhaps the answer to this lies in the concept of epistemic trespassing [31,32]. This term was coined by philosopher Nathan Ballantyne and describes the intrusion of experts into fields outside their own expertise. We have seen many examples of this during the COVID-19 pandemic. Particularly embarrassing to radiologists, Scott Atlas, a neuroradiologist, acted as COVID-19 advisor to Donald Trump during his presidency. Dr. Mehmet Oz, a cardiovascular surgeon and TV host, challenged Dr. Anthony Fauci, an accomplished expert in infectious disease and immunology, to a debate on COVID-19 “doctor to doctor”. A well-known anti-vaccine “doctor” in Australia has her doctorate in geology [33].

The composition of evidence review teams and some guideline panels suggests that epistemic trespassing is a factor in current guideline formulation. For example, the CTFPHC produces guidelines largely intended for use by primary care providers, predominantly physicians and nurses. Until recently, however, it was chaired by a psychologist. The CTFPHC breast screening guideline panel was chaired by nephrologists in both 2011 and 2018, and a chiropractor was on the knowledge tools team for the 2018 guideline. There was, however, no breast surgeon, radiologist, technologist, physicist, pathologist, oncologist, or patient on these teams. The main opportunity for input from content experts was an emailed form, similar to that provided to all external stakeholders. There was no opportunity for dialogue or teaching by content experts. The urologists involved with the 2014 CTFPHC prostate guideline were so dismayed at the CTFPHC consultation process that they resigned in protest.

In my conversations, with patients and even referrers, almost all of them are surprised to learn that the panels that form guidelines exclude the very experts they trust with their specialized healthcare. I suspect most people make the natural and trusting assumption that content experts make significant contributions to their healthcare guidelines. While the credentials of the authors of the CTFPHC guidelines are not hidden, they are not openly disclosed. The names of the authors of each guideline are provided, but their areas of expertise are not visible unless one specifically searches for their credentials. One could say that the lack of content expertise is hidden in plain sight.

## 5. Conflict of Interest (COI)

What is the reason for this counterintuitive guideline panel composition and lack of fulsome expert consultation? The stated reason seems to be an avoidance of conflict of interest (COI) [34,35]. There is an assumption that content specialists would try to boost their own incomes by influencing guidelines. When asked about the experts’ signatures on an open letter rebutting the 2018 breast screening guideline, the then-chair of the CTFPHC said, “They earn a living carrying out imaging services, and some also earn income through their work with companies that produce imaging equipment.” [36]. The news report did not mention any evidence-based rebuttal to the many points made in opposition to the breast screening guideline, however. This is an example of a logical fallacy known as *ad hominem*, in this case attacking the motivation of the speaker and ignoring the substance of the argument.

While COI is an important concern, particularly in the case of industry-sponsored research, it is far less pertinent to practising Canadian medical specialists. Many, if not most, Canadian medical specialists are overwhelmed with waitlists [37] throughout their careers and are unlikely to boost income with screening. In some cases, such as serologic screening for prostate and liver disease, the specialist physician has no direct financial COI at all.

Unfortunately, these unsubstantiated accusations of specialist physician COI lead to exclusion of content expertise. As we have seen with the continued use of CNBSS for guidelines, however, this is detrimental to the appropriate determination of scientific rigour. In fact, the implication of COI has specifically been used to dismiss valid concerns by experts, such as the excess deaths in the CNBSS screening arm [18].

I posit that in a single-payor healthcare system, the largest financial COI is that of the payor. In Canada, this is the government, which also happens to fund the CTFPHC via the Public Health Agency of Canada (PHAC). Screening programs are expensive and create further downstream expenses. It is understandable that minimizing screening recommendations would be a desirable guideline outcome for the healthcare payor.

## 6. Lack of Accountability

In April 2019, when asked by NDP Health Critic, Don Davies, to halt the use of the 2018 CTFPHC breast screening guideline, the federal Health Minister at the time, Ginette Petitpas Taylor, absolved the ministry of any responsibility, stating, “While the government provides its support to the Task Force to the breast cancer screening work group [sic] its decision was totally done independently. As such these are not official government guidelines” [38]. This statement was repeated almost verbatim by the Health Minister’s Parliamentary Secretary a few weeks later [39].

When asked about the news regarding the eyewitness accounts of misallocation of patients during randomization of CNBSS, PHAC issued a statement indicating that it provides funding to the Task Force and referred to the body as being an “arms-length from the government” [40], but took no further responsibility for the CTFPHC recommendations.

The current co-chair of the CTFPHC, when asked about the same eyewitness accounts, indicated that the group conducts “rigorous, detailed evidence reviews to formulate guidelines” and did not indicate that any further reviews would be performed, even in light of the new information [40].

The CTFPHC claims that its guidelines are ranked among the best in the world [40], but this warrants a closer inspection. A guidance statement and quality review of breast screening guidelines, authored by a group of guideline methodologists [41], failed to acknowledge that GRADE and AGREEII were not appropriately applied to the CTFPHC guideline. Despite completely excluding all modern observational evidence from the analysis of screening benefits and excluding any genuine consultation with content experts, the CTFPHC guideline scored well in this analysis. Guideline methodologists assess the quality of guidelines without the benefit of content expert input nor outcomes analyses, much like “marking each others’ homework”.

To whom is this publicly funded government agency accountable? It would appear that CTFPHC answers to no one.

Why might the government have set up an unusually unaccountable body to develop healthcare guidelines? As mentioned above, there is a large financial cost to screening, both directly and indirectly. Guidelines can be used to help control healthcare costs, and, ideally, good guideline recommendations will balance appropriate safe health care and judicious use of resources. Structuring a guideline body to be unaccountable, however, removes this balance and allows its recommendations to stand for years without correction of errors. There is another benefit to the arm’s-length status, however. According to National Cancer Institute Cancer Intervention and Surveillance Modeling Network (CISNET) modelling, 400 women may die each year as a result of the CTFPHC recommendation against screening women in the 40–49 age group [42]. Arm’s-length status may protect both PHAC and the Health Ministry from responsibility for these avoidable deaths.

## 7. Casting Doubt

When the rest of the evidence converges on the conclusion that screening saves lives, even for women aged 40–49, why continue to include the poorly performed outlier study in evidence analyses? One can certainly speculate that there is strong motivation to perpetuate the use of studies such as CNBSS. The outlier creates doubt around the benefit of screening women 40–49 and keeps the mammography screening controversy alive. In fact, the various techniques used to challenge the benefits of mammographic screening have been extensively discussed by Dr. Daniel Kopans in his analyses [43,44].

Have we seen this pattern of perpetuating doubt for financial benefit in the past? In fact, this strategy is known as “manufactured doubt” and has been employed for decades by large organizations [45,46]. In its typical form, it is used by industry to delay regulation by creating doubt about whether evidence converges on a particular outcome. It was famously used by the tobacco industry to delay regulation for decades, while the industry continued to reap billions of dollars of profits. Other examples include the opiate, silicates, talc, diesel, alcohol, and sugar industries. Doubt is manufactured by stressing outlier studies (such as CNBSS), cherry-picking data (such as excluding all observational data), and many other methods.

Strategies for manufacturing doubt are well documented [47], as many of the above-mentioned industries have undergone scrutiny and even litigation for these practices. The following is a selection of known strategies employed to manufacture doubt, listed in the linked article https://ehjournal.biomedcentral.com/articles/10.1186/s12940-021-00723-0 (accessed on 26 May 2022). These have been correlated to examples of their use by the CTFPHC and other critics of screening. Keep in mind that the strategies were written with large commercial industries in mind, and the wording may not be fully applicable to government and screening scenarios. Additionally, I limit most of my examples to breast screening recommendations.

**1. Attack study design**—Characterization of any studies that favour screening as flawed, frequently using CNBSS study as a comparator [48,49].

**2. Misrepresent data**—Cherry-picking or diluting the evidence by pooling poor- and good-quality studies in meta-analyses and evidence review [23,50,51]. Continuing to include CNBSS is an example of this. Another example is also noted in the prostate screening literature, mentioned later. Overestimations of overdiagnosis [4,51,52] are also used to create fear and discourage screening.

**3. Suppress incriminating information**—Observational studies, many of which are more modern than the RCTs, demonstrate a large degree of effectiveness. These are, however, excluded from the evaluation of the benefits of screening mammography in CTFPHC analysis [23]. Despite this, observational studies and even questionnaires are permitted in the evaluation of harms.

**4. Contribute misleading literature**—The CTFPHC performed a review of women’s values questionnaires [53], interpreted to suggest women would not want to screen, even though the questionnaire review demonstrates that women do desire screening

**5. Host conferences or seminars**—In 1997, the National Cancer Institute held a Consensus Development Conference of the National Institutes of Health on “Breast Cancer Screening for Women Ages 40–49”. Minority opinion was ignored, and the decision not to recommend screening for this age group was called “unanimous” [54].

**6. Blame other causes**—In the case of screening, rather than blame, benefits are attributed to other causes, particularly modern treatment [4,49,51].

**7. Invoke liberties/censorship/overregulation**—The recommendation not to screen women aged 40-49 is couched as “shared decision-making” [4], even though the CTFPHC recommendations result in limitation of the option to screen women aged 40-49 in many jurisdictions.

**8. Define how to measure outcome/exposure**—The CTFPHC assesses mortality benefits only, ignoring well-documented non-mortality benefits associated with earlier diagnosis, such as decreased severity of treatments, as well as lower incidence of long-term complications, such as lymphedema in screened populations [55].

**9. Pose as a defender of health or truth**—The CTFPHC emphasizes harms and minimizes benefits, stressing anxiety, biopsies, and exaggerated overdiagnosis rates. While the recommendations appear to put the patient’s emotional health first, they are paternalistic and represent a false equivalency in comparison with unnecessarily delayed diagnoses.

**10. Obscure involvement**—The unaccountable structure of the CTFPHC falls into this category.

**11. Normalize negative outcomes**—The CTFPHC stresses a lack of evidence of improvement in all-cause mortality (difficult to prove considering a relatively small proportion of the population dies of breast cancer [49,56]), minimizing the mortality benefits. This implies that excess deaths among non-screened women are acceptable. Additionally, the false equivalency of the potential harms (anxiety, biopsy, overdiagnosis) over the potential benefits of screening (lower likelihood of dying of breast cancer among those screened) normalizes avoidable breast cancer deaths.

**12. Attack Opponents (scientifically/personally)**—*Ad hominem* attacks on the motivation of dissenters, discussed earlier.

**13. Abuse of credentials**—Epistemic trespassing by non-content-experts, discussed earlier.

## 8. Broader Problems

I have largely emphasized the problems with the 2018 CTFPHC breast cancer screening recommendations, but similar problems exist within many of the other major extant CTFPHC guidelines. In a personal correspondence, a prominent urologist mentioned inappropriate handling of prostate screening evidence for the 2014 guideline.

“There is a precise analogy [to CNBSS] in the prostate cancer field, the PLCO study [57] of PSA screening. 85% contamination in the control arm and 15% non-compliance in the study arm (this is documented and published) resulted in no difference in the proportion tested, and therefore no mortality difference between the 2 arms. The other large scale study, ERSPC (European Randomised Study of Screening for Prostate Cancer) [58], was strongly positive. The task force looked at the 2 studies, noted one was positive and one negative, and concluded that therefore no convincing evidence of benefit.

We pointed out the flaw in their reasoning with our ‘stakeholders comments’ in 2014 and we received no response from the task force, and no evidence that they took our comments into account.

Dr. Laurence Klotz, MD, FRCSC, CM
Professor of Surgery, University of Toronto
Sunnybrook Chair of Prostate Cancer Research
Chairman, World Urologic Oncology Federation
Chairman, SI (Stability Index) UCare Research Office9
Chairman, Canadian Urology Research Consortium
Sunnybrook Health Sciences Centre”

Again, this indicates the pooling of poorly performed and well-performed research, creating doubt. Additionally, this demonstrates the lack of meaningful dialogue with highly qualified content experts. The use of the term “stakeholder” [59] is prejudicial, implying a material interest, or “stake”, in the guidelines, rather than professional interest and a role as expert advisors. The term “topic advisor” is preferable and is used in the NICE UK methodology [60].

In fact, multiple other prominent specialists and specialist societies have written rebuttals to the CTFPHC guidelines, many of which are evidence-based [61,62,63,64,65,66,67,68,69] (Supplementary Materials).

## 9. CTFPHC and the Suppression of Science

Is there any evidence that the government would deliberately suppress science? In fact, the Harper government did exactly that in the late 2000s. Climate change and environmental scientists were muzzled, and environmental research was inhibited, culminating in a 2012 protest on Parliament Hill, nicknamed the Death of Evidence March [70,71]. Climate change and environmental science have an impact on development of fossil fuels and thus the Canadian economy. During approximately the same time period and under the same federal government, the current structure of CTFPHC was initiated in 2010 [72].

## 10. Suggestions for Reform

The lack of expert guidance in the performance of evidence review and the formation of guidelines is problematic. This requires urgent reform, but CTFPHC requires a robust accountability structure for any reforms to take place. As it currently stands, the lack of expert guidance constitutes a breach of the public trust. The public should insist on fundamental reform to the structure of the CTFPHC. A new national guidelines body should be formed with appropriate oversight and accountability built in.

While COI is of serious concern, practising Canadian healthcare practitioners should not be conflated with “product defence” and other industry-funded experts. COI should be acknowledged for both content experts and for government agencies’ funding guidelines. COI should not, however, outweigh expertise and clinical experience. Ad hominem attacks on motivation should be avoided.

Any CTFPHC guidelines formed without fulsome expert guidance, particularly if Canadian content experts have provided evidence-based rebuttals, should be suspended from use pending content expert review and, if necessary, revision. In the interim, many national specialty societies have their own guidelines, which can be substituted for suspended CTFPHC recommendations.

Full disclosure of the credentials of personnel involved in evidence review and guideline formation is required for rebuilding trust in the processes.

Process transparency should be emphasized, and satisfaction surveys of panel members should be a mandatory element of guideline quality assessment. A tool such as PANELVIEW [73] could be adapted to this purpose.

Guideline quality should not only be evaluated based on adherence to guideline methodology, but also by outcomes. Following the USPSTF recommendation against PSA screening in 2012, metastatic prostate cancer increased, as predicted by modelling [74]. Outcomes follow-up should be mandatory following guideline recommendations, and this should be used to define guideline quality, rather than self-referential adherence to methodologies, which, as we have seen, may be misapplied or misrepresented.

Ethicists should be involved in the restructuring process of the CTFPHC, the formation of guidelines, and ongoing oversight of methodological processes. The Precautionary Principle [75] should be employed in all decisions that impact the well-being and lives of the population.

Where costs and other resource limitations are factored into guideline recommendations, this should be clearly disclosed. Science should not be manipulated to accommodate budgetary concerns.

## 11. Conclusions

The ongoing use of the flawed CNBSS is the natural consequence of significant systemic problems with the application of guideline methodology and, in Canada, with the unaccountable structure of the CTFPHC. While the practice of medicine requires close adherence to evidence, common sense and clinical judgment are the lenses through which evidence must be filtered. The evidence-based movement has been criticized, even by its proponents, calling for a “return to real evidence based medicine”, including “increasing depth of knowledge and sensitivity to context when applying rules” [76].

Making medical recommendations outside one’s area of specialty training is not accepted in clinical practice and should not be accepted in the formation of guidelines. Guideline oversight and methodology reform are required to provide appropriate expertise in guideline formulation. As a result of specialists’ career-long waitlists and resultant minimal COI, Canada is well positioned to produce excellent guidelines. To achieve these improvements, however, clinicians and patients must advocate for fundamental reform to guideline practices.

## Supplementary Materials

The following supporting information can be downloaded at: https://www.mdpi.com/article/10.3390/curroncol29060313/s1. References [77,78,79,80,81] are cited in the Supplementary Materials.
