# Supplementary material for: How Did CNBSS Influence Guidelines for So Long and What Can That Teach Us?
Source: Curr Oncol. 2022 May 30;29(6):3922–32. doi: 10.3390/curroncol29060313 (PMC9221595; doi:10.3390/curroncol29060313)
Supplement: Supplementary file 1 [file curroncol-29-00313-s001.zip › curroncol-1677133-supplementary.pdf]

Press release

To: **Canadian Urological Community**
October 27, 2014

Dear Colleague,

As you are likely aware, the Canadian Task Force on the Preventive Health Care (CTFPHC) (formerly the Canadian Task Force on the Periodic Health Examination, CTFPHE) has recently produced a guideline on prostate-specific antigen (PSA) screening. Their recommendations, directed at clinicians and policy-makers, apply to all men without a previous diagnosis of prostate cancer. They are as follows:

1. For men aged less than 55 years, they recommend not screening for prostate cancer with the PSA test. (**Strong** recommendation; low-quality evidence.)

2. For men aged 55–69 years, they recommend not screening for prostate cancer with the PSA test. (**Weak** recommendation; moderate-quality evidence.)

3. For men 70 years and older, they recommend not screening for prostate cancer with the PSA test. (**Strong** recommendation; low-quality evidence.)

The CTFPHC summary states: "There is (a) no evidence that PSA screening reduces overall mortality among men of any age; (b) conflicting evidence suggesting a small and uncertain potential reduction in prostate cancer mortality among men aged 55–69 years; (c) no convincing evidence of a reduction in prostate cancer mortality in any other age group; and (d) consistent evidence that screening and active treatment lead to harm."

PSA testing is controversial, and these recommendations are not dissimilar to the US Preventive Services Task Force position. However, many clinicians in the prostate cancer field anticipated a more positive recommendation given the following:

- the Level 1 evidence of a reduction in prostate cancer deaths seen in randomized Phase III trials,[1]

- the 45% reduction in deaths due to prostate cancer in Canada since 1995, and

- the widespread adoption of active surveillance for low-risk disease in Canada[2]

Process employed by the Task Force: The Task Force sought to determine the balance of risks and benefits of PSA screening in developing these recommendations. Importantly, the members of the Task Force did not include any clinician or scientist with a background in prostate cancer. While the process of developing the recommendations included an extensive literature review, the Task Force focused on the results of prospective randomized trials, and, in our view, discounts a great deal of evidence that, for those of us who work in the field, is compelling. This is summarized below.

Our Concerns: The recommendation advises strongly against PSA testing in men under age 55, acknowledging that this is based on poor quality evidence. This recommendation does not incorporate the compelling population-based data from Sweden demonstrating that baseline PSA is strongly predictive of the future risk of aggressive prostate cancer 20 to 30 years later, thus warranting less frequent screening in men with a low PSA.[3]

1. The Task Force comments that the randomized trials do not show a decrease in overall mortality. This is misleading to the reader because none of the screening trials were powered to demonstrate a decrease on overall mortality. For example, a trial designed to have 80% power to detect a 50% decrease in cancer mortality at 10 years, from 1 to 0.5%, in a population with an overall 20% mortality at 10 years, would have less than 10% power to detect a difference in all-cause death. Thus the lack of an overall mortality reduction should not be considered a criticism.
2. The document acknowledges that two of the higher quality trials found a reduction in prostate cancer-specific mortality, whereas four lower quality trials found no difference between the screening and control groups. The contamination in the PLCO trial, which has been reported to be as high as 85%, among other flaws, means that PLCO should not be considered equivalent to the ERSPC study. In other words, the Task Force observed that the strongest evidence revealed a reduction in prostate cancer death; however, the recommendation states there is "conflicting evidence suggesting a small and uncertain potential reduction in prostate cancer mortality." The statement acknowledging a mortality reduction from screening observed in the robust trials is at odds with the statement in the final recommendation that there is no clear evidence of a mortality reduction.
3. The review understates the benefit of screening, which it states as 1.28 deaths avoided per 1000 men screened. The published report from the Goteborg randomized trial is that with 14 years of follow–up, the number needed to diagnose for each death avoided is 12,[4] and in an analysis of healthy screened patients in PLCO, it is 5. The adjusted mortality reduction (corrected for non-compliance) in ERSPC was 27% at 13 years, while the Task Force quoted the unadjusted rate of 21%.

4. Evidence for a decrease in metastatic disease is also important to patients and was not included.[5] Further, the mortality curves in ERSPC and Goteborg continue to diverge with longer follow-up. While a well-founded expectation of more benefit being demonstrated with longer follow-up should not drive current recommendations, it is reasonable that it influences the strength of these recommendations.
5. The unsubstantiated claim that the reduction in mortality is unlikely due to screening and more likely due to advances in treatment is contrary to published evidence. Epidemiological modelling studies consistently ascribe 40% to 75% of the reduction in mortality to screening,[6] and only 20% to 33% to changes in treatment.
6. Active surveillance has been widely adopted in Canada. This was not mentioned in the document. Clearly the widespread use of surveillance for low-grade disease in Canada is relevant.

In conclusion, the best trials available to date, which are still in progress, have demonstrated that screening reduces prostate cancer death by 21% to 44%. To recommend against screening because “Available evidence does not conclusively demonstrate that screening with the PSA test will reduce mortality from prostate cancer” is misleading and reflects errors of fact, omission, interpretation, and statistics.

PSA screening has had a major impact on prostate cancer mortality, but carries with it the risk of harm to patients who are unlikely to benefit. In our view, the following recommendations are more appropriate for a Canadian population.

1. Avoid PSA testing in men with little to gain. After appreciating the potential risks and benefits, those men who do decide to have a PSA and have a low value (<1.0 at baseline) should be tested infrequently, about every 5 years. Men with less than a 15-year life expectancy (typically over age 70) should not be screened unless they had a high PSA previously. Men whose PSA is above the median for their age but below the biopsy threshold should be counselled for more regular screening and risk assessment.
2. Digital rectal exam (DRE) has value for the detection of many anal and rectal problems, as well as prostatic abnormalities in addition to prostate cancer. DRE should continue to be performed as a routine part of the periodic health exam.
3. Do not treat men with low-risk prostate cancer, or older men with intermediate-risk prostate cancer, who are not likely to benefit from treatment.

A weak recommendation against widespread screening for men aged 55-69 suggests that there is uncertainty in the value of PSA, and hence a discussion between patient and care provider should take place to weigh the pros and cons.

This leaves the door open for clinicians to offer PSA testing to patients at increased risk, or those who wish to reduce their likelihood of prostate cancer mortality. Further, this recommendation is acknowledged to be a document in evolution; the Task Force process includes a review of the recommendation should new data emerge. We are confident that that the Task Force will eventually revise this recommendation as ERSPC matures further, as new tests reduce the requirement for biopsy and improve risk stratification, and as the morbidity of treatment improves with new minimally invasive approaches.

Signed: Laurence Klotz, Rodney Breau, Joseph Chin, Antonio Finelli, and Martin Gleave on behalf of the Canadian Urological Association

Laurence Klotz, Division of Urology, Sunnybrook Health Sciences Centre, University of Toronto, Toronto, ON

Rodney Breau, Division of Urology, Department of Surgery, The Ottawa Hospital; and the Ottawa Hospital Research Institute, Ottawa, ON

Joseph Chin, Division of Urology, Schulich School of Medicine and Dentistry, Western University, London, ON

Antonio Finelli, Division of Urology, Department of Surgical Oncology, University Health Network, Toronto, ON

Martin Gleave, The Vancouver Prostate Centre and Department of Urologic Sciences, University of British Columbia, Vancouver, BC

Conflict of interest statements available upon request.

Contact information:
Tiffany Pizioli
CUA Executive Director
185 Dorval Ave., Suite 401
Dorval, QC H9S 5J9
Cell: 514-813-8850

References

1. Schröder FH, Hugosson J, Roobol MJ, et al. Screening and prostate cancer mortality: results of the European Randomised Study of Screening for Prostate Cancer (ERSPC) at 13 years of follow-up. *Lancet* 2014 Aug 6. http://dx.doi.org/10.1016/S0140-6736(14)60525-0
2. Siegel R, Ma J, Zou Z, et al. Cancer statistics. *CA Cancer J Clin* 2014;64:9-29.
3. Lilja H, Cronin AM, Dahlin A, et al. Prediction of significant prostate cancer diagnosed 20 to 30 years later with a single measure of prostate-specific antigen at or before age 50. *Cancer* 2010;117:1210-9.
4. Hugosson J, Carlsson S, Aus G, et al. Mortality results from the Göteborg randomised population-based prostate-cancer screening trial. *Lancet Oncol* 2010;11:725-32.
5. Schröder FH, Hugosson J, Carlsson S, et al. Screening for prostate cancer decreases the risk of developing metastatic disease: findings from the European Randomized Study of Screening for Prostate Cancer (ERSPC). *Eur Urol* 2012;62:745-52.
6. Etzioni R, Gulati R, Tsodikov A, et al. The prostate cancer conundrum revisited: treatment changes and prostate cancer mortality declines. *Cancer* 2012;118:5955-63.

Suggested recent and outstanding readings on this topic:

Wilt TJ, Scardino PT, Carlsson SV, et al. Prostate-specific antigen screening in prostate cancer: perspectives on the evidence. *J Natl Cancer Inst* 2014;106(3).
An excellent and balanced summary of the evidence for and against screening by a member of the USPSTF and several well-known proponents of screening.

Etzioni RD, Thompson IM. What do the screening trials really tell us and where do we go from here? *Urol Clin North Am* 2014;41:223-8.
An analysis by an epidemiologist and a urologist; a very balanced perspective.

Hayes JH, Barry MJ. Screening for prostate cancer with the prostate-specific antigen test: a review of current evidence. *JAMA* 2014;311:1143-9.
An epidemiologist and a clinical epidemiologist evaluate the evidence for and against screening.

Schröder FH. ERSPC, PLCO studies and critique of cochrane review 2013. *Recent Results Cancer Res* 2014;202:59-63.
The ERSPCI PI reviews the evidence and critiques the Cochrane review on the subject.

Vickers A, Carlsson S, Laudone V, et al. It Ain't What You Do, It's the Way You Do It: Five Golden Rules for Transforming Prostate-Specific Antigen Screening. *Eur Urol* 2014;66:188-90.
A practical and rational approach to screening by two epidemiologists and a biochemist.
